# Impact of smoking on dental implant: A review

**DOI:** 10.6026/9732063002001750

**Published:** 2024-12-31

**Authors:** K Sohith Reddy, Snigdha Biswas, Sibani Sarangi, Akankasha Chaurasia, M. Pradeep Reddy, Ajimol Theresa Jose, Ritik Kashwani

**Affiliations:** 1 Department of Computational Biology, Saveetha School of Engineering, SIMATS University, Chennai, India; 2 Department of Periodontology and Implantology, BBD College of Dental Sciences, Lucknow, Uttar Pradesh, India; 3 Department of Periodontology, Hitech Dental College and Hospital, Bhubaneswar, India; 4 Department of Oral and Maxillofacial Surgery, Jaipur Dental College, Maharaj Vinayak Global University, Jaipur, India; 5 Department of Prosthodontics and Crown and Bridge, Buddha Institute of Dental Sciences, Patna, Bihar, India; 6 Department of Prosthodontics & Implantology Faculty Of Dentistry, AIMST University, Kedah, Malaysia; 7 Private Practitioner, School of Dental Sciences, Sharda University, Greater Noida, India

**Keywords:** Implants, osseointegration, smoking, titanium

## Abstract

Dental implants are the most innovative strategy for substituting a missing tooth. Smoking has been identified as a significant risk factor impacting the success rates of dental implants. This study explores the correlation between tobacco use and implant failure focusing on the physiological mechanisms involved. Smoking impairs osseointegration, the critical process where the implant integrates with the alveolar bone due to reduced blood flow oxygenation and angiogenesis caused by nicotine and carbon monoxide. Smokers exhibit higher rates of implant failure compared to non-smokers with compromised bone quality and delayed wound healing as contributing factors. Studies suggest that smoking adversely affects implant stability, particularly during the critical early healing phase. This review highlights the biological mechanisms and clinical implications of smoking on dental implant outcomes.

## Background:

Implants are biocompatible surgical devices that need to be inserted into the jawbone in order to replace a lost tooth's root. They provide a solid foundation for fixed or removable prosthetic teeth that are designed to mimic natural teeth in terms of appearance as well as function. Osseointegration, a mechanism that occurs when an implant merges with the structure of the bone periodically, affirms the durability and effectiveness of it over the course of time [1]. Smoking has been identified as a significant risk factor for dental implant failure. The deleterious effects of tobacco use on oral health, particularly concerning dental implants, are well-documented. Nicotine and other harmful substances in cigarettes impair blood circulation, hinder osseointegration-the critical process by which implants anchor to the jawbone-and compromise immune responses, leading to increased susceptibility to infections such as peri-implantitis. Consequently, smokers exhibit higher rates of implant failure compared to non-smokers. A systematic review analyzing recent studies reported that smoking is associated with a higher incidence of dental implant failure, underscoring the need for smoking cessation to enhance implant success rates [2]. In present-day dental medicine, an assortment of implants of various kinds is utilized, individually customized to cater to the particular requirements of the patient. The type that is most prevalent is the endosteal implant, which functions as an adjunct root for single crowns or bridges, being embedded right into the jawbone Subperiosteal implants are often installed in individuals who are lacking sufficient bone density and are thus unable to take on bone augmentation. They are installed beneath the gum tissue but directly over the jawbone. Further form of implant is the zygomatic implant that anchors into the zygoma of the cheekbone so it offers support in cases where the upper jawbone has suffered notable deterioration. Furthermore, smaller-diameter micro implants can be used for stabilizing prostheses [3]. The three elementary components of a dental implant are the prosthesis, also known as the crown, the abutment and the implant body, sometimes referred as the fixture.

## Methodology:

A systematic search strategy was employed to review the literature on the impact of smoking on dental implants. The search was conducted across databases including PubMed, Scopus, Cochrane Library and Google Scholar, using a combination of MeSH terms and keywords such as "dental implants", "smoking", "tobacco", "implant failure", "peri-implantitis" and "complications". Boolean operators were used to refine the search, ensuring comprehensive coverage of the topic. Studies published between [insert years] were included, focusing on systematic reviews, meta-analyses, clinical trials and observational studies in peer-reviewed journals. The search was restricted to English-language articles with full-text availability. Inclusion criteria targeted studies assessing the effects of smoking on dental implants, while studies unrelated to the topic, case reports, non-peer-reviewed articles and animal or in vitro studies were excluded. Two independent reviewers screened titles and abstracts for relevance, with discrepancies resolved through consensus. Data were extracted on study design, population characteristics, outcomes such as implant success rates and complications and key findings. The quality of the studies was assessed using appropriate tools such as the Newcastle-Ottawa Scale and Cochrane Risk of Bias tool and systematic reviews were evaluated using the AMSTAR-2 checklist.

## Review:

Through several procedures, smoking has adverse effects on dental implant's success alongside overall oral health. By reducing blood flow to the gingiva, it degrades periodontal health by impairing the tissues' capacity to fight off infection and repair adequately. Periodontal disease can arise from this; it is marked by loss of alveolar bone, periodontal pocket formation and gingival inflammation. Smoking elevates the risk of peri-implantitis, a condition characterized by inflammation which impacts tissues around dental implants and may result in implant failure and bone loss. Also, smoking interferes with normal bone remodeling and suppresses the immune system, which impedes the "osseointegration" process and raises the risk of implant problems and failure [4].

From meta-analyses, the overall success rate of dental implants is around 95% for non-smokers. On the contrary, smokers have much lower success rates that vary from 85% to 90%, contingent upon when or for how long they smoke. Data also shows as compared to light or moderate smokers, heavy users-those who smoke more than 20 cigarettes a day-generally have less favourable results. Significantly, quitting smoking at the time of the treatment may raise the chances of success; as to some studies, patients who quit smoking prior or following surgery had an outcome of about 92%.in bone resorption and implant failure. Additionally, smoking impairs the osseointegration process-the integration of the implant with the surrounding bone-by disrupting normal bone remodelling and diminishing the immune response, further increasing the likelihood of implant complications and failure [5].

## Discussion:

Dental implants offer an assortment of advantages (Table 1). Titanium is employed for the production of implants, which have an exceptionally long lifespan-many decades or longer with good upkeep. In contrast to alternate options, implants provide a more visually appealing result as they seem and feel like natural teeth. By encouraging bone development, implants assist in preserving bone density and therefore avert bone loss that often follows tooth loss. They can be utilized for eating normally, speaking and biting with minimal discomfort as these function like an actual tooth. Implants retain the integrity of adjacent natural teeth since they do not demand modifications, as opposed to bridges. Implants will improve general oral hygiene since they are easier to maintain and clean [6], although there are many advantages to dental implants, there are also limitations to be taken into account. Dental implants need to be placed surgically, which entails risks like infection, damage to adjacent teeth or nerves and specifically in cases of upper jaw implants, sinus issues. During the healing process, this could be uncomfortable or problematic. The procedure by which the implant fuses with the bone is referred to as osseointegration and it typically takes three to six months. Patients might require temporary restorations during this time, which could result in longer treatment periods than with other dental alternatives like bridges or dentures. Typically, dental implants are more costly than alternative restoration options as bridges or dentures. Prior to getting implants, patients with little jawbone density might need additional procedures including sinus lifts or bone grafting. Though having a high level of success, dental implants can fail for a variety of reasons, including inadequate osseointegration, inflammation around the implant, or severe stresses from bruxism. Implants need to be carefully cleaned in order to avoid peri-implant infections like peri-implant mucositis or peri-implantitis [7]. Both oral health and several kinds of external factors influence the rate of success of implants; they are illustrated in the accompanying [8].

## Impact of smoking on implant success:

Smoking interferes with the success of dental implants by impairing immunological response, reducing blood flow, reducing bone integration, increasing the risk of infection and poor oral hygiene [9]. It hampers the integration and healing of bones. The balance between the formation and resorption of bone is disrupted by the compounds in cigarettes, notably nicotine, This imbalance favours increased bone resorption over formation, leading to a compromised bone matrix and negatively affecting the implant's stability and integration. This has an impact on the process of osseointegration. By modulating the expression of osteogenic markers such alkaline phosphatase and osteocalcin, nicotine limits their growth and activity. As a result, the area around the implant grows less bone [10]. Tobacco smoke causes vasoconstriction, leading to reduced blood flow. This decreases the oxygen and nutrient supply to the bone and surrounding tissues, impeding healing and increasing the risk of implant failure. Smoking leads to reduced salivary flow and changes in the composition of saliva, which can affect oral hygiene. Poor oral hygiene increases the risk of plaque accumulation and subsequent bacterial infection around the implant, further compromising implant success [11].

By modulating immunological cell activity and cytokine production, nicotine alters the inflammatory response. It can change the inflammatory milieu locally, which is necessary for osseointegration to occur properly and for healing to proceed. Inflammation brought on by nicotine use may cause the bone surrounding the implant to grow more slowly or not at all. The effects of nicotine on the immune system may make a person more vulnerable to illnesses. A heightened susceptibility to infection may lead to peri-implantitis, an inflammatory disorder that impedes osseointegration even further [12]. Combining techniques to increase the chance of osseointegration and reduce complications are a way to mitigate the detrimental impact of smoking on dental implants. Implant success rates can be considerably raised by providing resources and guidance for quitting smoking, such as medication, counselling and nicotine replacement therapy [13]. An in-depth assessment of the individual's overall wellness and smoking history could help in establishing the best course of action. Pre-operative diagnostics and imaging can guarantee that possible problems are taken care of prior to implant Modern surgical methods and supplies can help lessen some of the harmful impacts of smoking. Methods that may enhance bone quality and facilitate successful osseointegration involve the application of growth hormones or bone transplants [14]. Strict adherence to post-operative care instructions is essential. This includes using antimicrobial rinses, keeping scheduled appointments and maintaining excellent oral hygiene. Smokers should be advised to maintain stringent oral hygiene practices, such as brushing, flossing and using antibacterial mouthwashes [15]. Good oral hygiene helps reduce plaque accumulation and the risk of peri-implantitis. Medication or therapies may be urged to support healing and reduce inflammation. If the possibility of infection is substantial these may include the use of local or systemic antibiotics. Using implants with specialized coatings or materials designed to improve integration in compromised conditions may be advantageous. Such implants may offer better results in smokers [16].

## Conclusion:

Smoking adversely impacts the success rates of dental implant by impairing osseointegration and peri-implant tissue stability. The vasoconstrictive effects of nicotine and cytotoxic properties of tobacco byproducts reduce vascularization, delay wound healing and compromise bone remodeling process. Nicotine-induced vasoconstriction and oxidative stress exacerbate inflammatory responses around the implant site, creating an environment conducive to bacterial colonization and biofilm formation. Additionally, smoking increases susceptibility to peri-implantitis marginal bone loss and implant failure due to its negative influence on immune response and microbial balance. Smoking cessation improves blood flow, promotes tissue regeneration and reduces the likelihood of complications. Dentists and healthcare professionals must prioritize patient education regarding the detrimental effects of smoking on oral health and provide robust support for cessation efforts. Consequently, smoking cessation is strongly advised as a part of pre-operative and post-operative care to mitigate risk and enhance long-term implant survival and functionality.

## Figures and Tables

**Table 1:** The success rate of dental implants is influenced by both oral health and various external factors.

**Oral health factors**	**External factors**
Bone Density: Adequate bone density is crucial for the stability and integration of implants. Poor bone quality can lead to implant failure.	Impact on Healing: Smoking impairs blood flow to the gums and bone, which can hinder healing and increase the risk of complications.
Bone Volume: If there's insufficient bone, bone grafting or other procedures may be needed.	Increased Risk of Infection: Smokers are at a higher risk for infections, which can affect Implant success.
Periodontal Disease: Active gum disease can undermine the success of implants by compromising the surrounding bone and soft tissue.	Diabetes: Poorly controlled diabetes can affect the healing process and increase the risk of implant failure. Good glycaemic control is important.
Gingival Health: Healthy gums are essential for supporting the implant and ensuring long-term success.	Osteoporosis: Conditions that affect bone density can impact the success of implants
Plaque Control: Effective brushing and flossing are crucial to prevent plaque Build-up, which can lead to peri-implantitis.	Impact on Bone Metabolism: Some medications, such as bisphosphonates, can affect bone metabolism and healing.
Regular Dental Visits: Routine check-ups help in early detection of issues that could affect implant success?	Interactions: Medications can also interact with other treatments or affect oral health, influencing implant success.
Positioning: Correct positioning of the implant in relation to adjacent teeth and anatomical structures is essential for functionality and aesthetics.	Bone Healing: Adequate nutrition is essential for bone healing and overall oral health.
	Bone Density Changes: As people age, bone density may decrease, which can affect the success rate of implants.
